# Research on the Participation of Chinese Sports Cultural Elite in Ice and Snow Sports

**DOI:** 10.3389/fpubh.2021.820765

**Published:** 2022-01-13

**Authors:** Ping Li, Wei Liu

**Affiliations:** ^1^School of Physical Education, Hua Qiao University, Xiamen, China; ^2^College of Humanities and Social Sciences, Xi'an Jiao Tong University, Xi'an, China; ^3^Department of Physical Education, Anhui Vocational and Technical College of Sports, Hefei, China

**Keywords:** mass sports participation, government policies, social mobilization, mobilization effect observations, sports

## Abstract

Governments have a responsibility to provide equal opportunities for sport and physical activity to all people of population. Chinese governments have issued many policies, such as “exhibition in the south, expansion in the West and East” of ice and snow sports to promote and stimulate the participation of the broad masses of the people. As a high-cost sport, the participants of ice and snow sports are usually socially elite groups. This study investigated the participation of cultural elite groups in ice and snow sports and investigated the social mobilization effect of ice and snow sports participation promotion policies by using binary regression and sequential regression models. The research shows that there are two different stages of one-time and continuous participation in the development of ice and snow sports in China. The one-time participation of ordinary people in ice and snow sports is mainly in response to the social mobilization of government policies. At the same time, it is positively correlated with site restrictions and knowledge of ice and snow sports. In the continuous participation group, gender, income, perception of ice and snow culture, and convenience near the site were highly positively correlated with consumption level. According to the results, low- and middle income people are less likely to participate in these activities because of their income. Therefore, this policy can increase inequalities.

## Introduction

Increasing physical activity levels is one of key component of the UN's 2030 Sustainable Development Goals. Investing in population based policies to promote sport and physical activity opportunities including walking, cycling, active recreation, and play can contribute directly to achieving many of the 2030 Sustainable Development Goals. So, policy actions on sport and physical activity can have multiplicative health, social and economic benefits, and will directly contribute to achieving SDGs (1). Governments have a responsibility to provide equal opportunities for sport and physical activity to all people of population. However, there are fewer opportunities for low and middle income settings. For this reason, policies must take into account the whole of population.

Chinese government, with its policies, seeks to increase level of physical activity. One of these policies is increasing people's participation in ice and snow sports. To realize the vision of “300 million people on ice and snow”, Chinese governments at all levels and their functional departments have issued policies to promote the participation of ice and snow sports. In 2016, the State General Administration of Sports, the National Development and Reform Commission, the Ministry of Education and the National Tourism Administration jointly issued notices on the development plan of ice and snow sports (2016–2025). In November of the same year, the State General Administration of sports issued the plan for the promotion and popularization of mass winter sports (2016–2020). In 2019, the general office of the CPC Central Committee and the general office of the State Council issued the notice the opinions on taking the 2022 Beijing Winter Olympic Games as an opportunity to vigorously develop ice and snow sports can clearly see that China has made great efforts to develop ice and snow sports with the help of Beijing's hosting of the Winter Olympic Games (2). In this year, the general office of the State Council issued the outline for the construction of a sports power, which proposed to “promote the strategy of” South exhibition, West Expansion and East advancement “of ice and snow sports and drive” 300 million people to participate in ice and snow sports “(2). On January 28, 2021, IOC President Bach told China's Xinhua News Agency: we can clearly point out that with “300 million people participating in ice and snow sports”, the history of world ice and snow sports will be divided by the Beijing Winter Olympic Games (2). Therefore, the Beijing Winter Olympic Games will become a milestone in the development of world ice and snow sports.

The development of ice and snow sports in middle-income China has important social significance. Ice and snow sports are a highly challenging and high-consumption project, which means that more people participating in the sport are social elites. In addition, as a project with low popularity and awareness in China, under the mobilization of national policies, highly educated social elites are more likely to accept, learn and consume. In other words, cultural elites are the pioneers of ice and snow sports participation.

The specific situation of people's participation in ice and snow sports, how to realize the participation effect, and whether the social mobilization effectiveness of the current policy is sustainable need to be discussed. Based on this, this paper will analyze the sustainable willingness of cultural elite groups to participate in ice and snow sports, examine the social internal causes of the development of ice and snow sports in China, and provide reasonable suggestions for more people to participate in ice and snow sports.

## Methodology

This study lists the cultural elite group as the survey object and intends to analyze the participation of cultural elite groups in ice and snow sports through three dimensions. First, the contingency table of cultural elites' response to the policy of “300 million people participating in ice and snow sports” and the contingency table of cultural elites' annual participation in ice and snow sports are analyzed in order to grasp the macro situation of cultural elites' participation in ice and snow sports from the survey data; Second, logit analysis of cultural elites' participation in ice and snow sports, determine the significant variables affecting their participation in ice and snow sports through regression model, and clarify the relevant factors promoting their participation in ice and snow sports; The third is to analyze the annual times of cultural elites' continuous participation in ice and snow sports, and determine the significant variable factors of this group's continuous participation in ice and snow sports through this kind of regression model.

These research data come from the research on the strategic value and promotion system of “South exhibition, West Expansion and East Expansion” of China's ice and snow sports, which is a national social science fund project of China, and the research on the implementation of “South exhibition, West Expansion and East Expansion” strategy of China's ice and snow sports and the improvement of implementation effectiveness in the next stage, which is entrusted by the winter sports management center of the State Sports Administration of China The network questionnaire of the research group. The network survey time is January 2021. To ensure the reliability of the network questionnaire, the following control measures are taken: first, the distribution object is controlled as the main highly educated object groups (mainly including scientific researchers in colleges and universities, college teachers and other highly educated object groups in society). Second, to avoid repeated answers, each IP address could only answer the questionnaire once during the network survey. Third, questionnaires with an answer time <1 min were eliminated, and some questionnaires that obviously did not answer carefully were eliminated through manual browsing in the later stage. A total of 326 samples were collected. After excluding missing values and outliers, 324 valid questionnaires were obtained, and 215 samples were selected for this study.

In particular, the data of this study are the survey conducted by the research team when informing the respondents of the purpose, which does not involve any ethics and privacy, and can be applied to the study of this project.

This paper uses questionnaire survey to collect data. In this paper, Stata 16.0 software was used to analyze the data, and logit and ologit models were used for estimation. Among them, the logit model is one of the discrete choice models. In this paper, a binary logit regression model is selected for the analysis of cultural elite participation. In the dependent variable, two values 1 and 0 (virtual dependent variable) are taken, that is, participation is yes or no. Ologit has become a sequential regression model. This paper converts the participation times into sequential data for analysis of the annual participation times of cultural elites and studies the factors that increase their continuous participation times through the sequential model.

### Variable

In terms of variable selection, we not only select according to the traditional sociological analysis variables but also design according to the preset research ideas and determine the result variables and independent variables in combination with the existing literature research, as follows:

#### Outcome Variable: Participating in Ice and Snow Sports and Now Participating in Ice and Snow Sports Every Year

In this study, two outcome variables, whether they have participated in ice and snow sports and the number of times they participate in ice and snow sports every year, were used to evaluate the social mobilization effect of 300 million people participating in ice and snow sports. The option of whether to participate in ice and snow sports is set to yes or no, and the values are 1 and 0 according to the two classification variables. Now, the options for the number of times to participate in ice and snow sports each year are set to 0, 1, 2, 3, and 4 times or more, and the values are assigned as 0, 1, 2, 3, and 4 according to the continuity variable.

#### Independent Variable: Population Sociological Characteristics + Social Sports Factors

The purpose of this study is to explore the social mobilization effect of 300 million people participating in ice and snow sports based on an empirical study of cultural elites. Therefore, the sociological characteristics of the population and social sports factors are selected as independent variables.

The sociological characteristics of the population mainly include gender, education and income. The first is the gender characteristics. Traditional ethical values still affect the value standards of Chinese women. It can be basically predicted from life experience that men prefer to participate in sports activities than women. In terms of gender, men = 1 and women = 0. The second is the characteristics of education. The scope of this study is the cultural elite. The selected category of cultural elites is personnel with a graduate degree or above. After converting the degree into the variable of years of education, it is 18. Income inequality has an important impact on the consumption behavior and social attitude of social members. The total income of residents includes personal wage income, business income, property income and transfer income. The income variable in this paper is: what was your annual income in the previous year? The options are set as below 100,000 yuan, 100,000–200,000 yuan, 200,000–300,000 yuan, 300,000–500,000 yuan and above 500,000 yuan. The values are assigned as 0, 1, 2, 3, and 4 according to the continuity variable.

The social sports participation factors of the crowd mainly select the cultural habits and the convenience of the venue. Bourdieu once pointed out that people's interests and hobbies exist in a certain “field and habits”. This study selects “like watching ice and snow competitions” and “understanding ice and snow sports” as explanatory variables. Among them, the options of “like watching ice and snow competitions” are set as: very dislike, dislike, general, like and very like, and are assigned as 0, 1, 2, 3, and 4 according to the continuity variable; The option of “understanding ice and snow sports” is set to very little understanding, little understanding, general, understanding and very understanding, and is assigned as 0, 1, 2, 3, and 4 according to the continuity variable. As the carrier of ice and snow sports, the convenience of ice and snow sports venues (pavilions) affects people's enthusiasm to participate in ice and snow sports activities. Therefore, this study takes ice and snow sports venues (pavilions) as an important independent variable. Therefore, “Is it convenient for you to go to the ice and snow venues near your residence?” To explain the convenience of ice and snow venues, this option is set to very inconvenient, inconvenient, general, convenient and very convenient. It is assigned as 0, 1, 2, 3, and 4 according to the continuity variable.

The three key independent variables of sports participation are consumption perception, policy cognition and participation times. First, due to the particularity of venues, climate and equipment, the consumption of ice and snow sports is higher than that of general sports. We choose” do you think the consumption of ice and snow sports are expensive?” to explain the consumption perception of ice and snow sports. The options are set to very expensive, relatively expensive, general, not too expensive and very cheap and are assigned as 0, 1, 2, 3, and 4 according to the continuity variable. Second, the social mobilization effect of ice and snow sports policy should first be the awareness of the policy. The research uses the question “have you heard of '300 million people participating in ice and snow sports”, the options are set to no and yes, and the values are 0 and 1 according to the two classification variables. At the same time, the question “when did you first participate in ice and snow sports?” is used to respond. The previous value is 02015, and the first time after 2015 is 1. Third, to investigate the continuity of the effect of social mobilization, this study sets the variable of “the number of times of participating in ice and snow sports every year”, which is set as 0, 1, 2, 3, and 4 times and is assigned as 0, 1, 2, 3, and 4 according to the continuity variable. The descriptive statistics of the above variables are shown in Table 1.

**Table 1:** Variable descriptive statistics.

**Variable**	**Frequency**	**Mean**	**Variance**	**Min**	**Max**
Gender	215	0.674	0.47	0	1
Education	215	18	0	18	18
Income	215	1.888	0.879	1	5
I like watching ice and snow games	215	2.502	0.748	1	4
Learn about ice and snow sports	215	2.056	0.835	0	4
Site convenience	215	1.349	1.121	0	4
Consumption perception	215	1.26	0.675	0	4
300 million people participate in ice and snow	215	0.693	0.462	0	1
Participated in ice and snow sports	215	0.526	0.501	0	1
Participation after 2015	215	0.269	0.445	0	1
Number of participants per year now	113	1.23	1.433	0	4

## Results

According to the descriptive statistics of the variables in Table 1, there is a large gap between the mean difference and the reasonable mean difference of four variables: income (1.888), site convenience (1.349), consumption perception (1.26) and participation after 2015 (0.269). Among them, the reason for the large difference in the mean value of income variables can be explained by the fact that the research groups are cultural elites, mostly belong to posts within the system, and the income is relatively stable and similar. There is a large difference in the mean value of site convenience. It can be assumed that most of the respondents are not very convenient from ice and snow activity sites (pavilions). There is a large gap in consumption perception. It can be assumed that this group thinks that the price of participating in ice and snow sports is expensive. If there is a large gap between the respondents and the average after 2015, it can be assumed that this group has a limited response to the policy.

### Descriptive Statistics of Participation in Ice and Snow Sports

The social mobilization effect of the policy of “300 million people participating in ice and snow sports” is first reflected in the degree of response. From the contingency table of policy response (Table 2), of the 215 samples investigated, 148 people have heard the saying of “300 million people participating in ice and snow sports”, accounting for 69% of the total; 58 people have participated in ice and snow sports after 2015, accounting for 26.9% of the total; Among the people participating in ice and snow sports in 2015, 50 of them have heard of “300 million people participating in ice and snow sports”, accounting for 86.2% of the people participating in ice and snow sports after 2015. It shows that cultural elite groups have played a positive role in participating in ice and snow sports.

**Table 2:** Contingency table of the response to the policy of “300 million people participating in ice and snow sports” by cultural elites.

**The first time to participate in ice and snow sports is after 2015**	**300 million people participate in ice and snow sports**		
	**Never heard of it**	**Yes**	**Total**
No	58	99	157
Yes	8	50	58
Total	66	149	215

Whether the social mobilization effect of cultural elites participating in ice and snow sports in the year is sustainable needs to be further discussed. According to Table 3 of the time series, the number of cultural elites participating in ice and snow sports in the years before and after 2015 was more than once. This is consistent with the research results of Sun (2019): at present, the penetration rate of the skiing population in China is only 0.87%, the conversion rate of the skiing population is low, and the average skiing population only skies 1.1 times a year (2). Among them, the participation of 0 times can be presumed to be an attempt under sports tourism, which shows that the objective investigation of the participation of cultural elite groups in ice and snow sports needs to be considered from two angles: one is the choice of participation, and the other is the continuity of participation. Because whether to choose may take into account the participation of “drainage” and “experience”, continuous participation is to test the “transformation” after participation.

**Table 3:** Contingency table of the number of times cultural elites participate in ice and snow sports each year.

**The first time to participate in ice and snow sports is after 2015**	**How many times do you participate in ice and snow sports every year?**						
	**Not involved**	**0**	**1**	**2**	**3**	**4 times or more**	**Total**
No	102	31	10	3	1	10	157
Yes	0	17	21	8	5	7	58
Total	102	48	31	11	6	17	215

### Yes or No: Logit Regression Model of Cultural Elites Participating in Ice and Snow Sports

The first dependent variable of this study is the dichotomous variable of whether they have participated in ice and snow sports. Therefore, dichotomous linear regression (logit) is used to analyze the impact of cultural elites' participation in ice and snow sports. Table 4 presents the estimation results of seven binary multiple linear regression (logit) equations: model 1 is the basic model, including two variables: gender and annual income. Models 2 to 7 add variables such as watching ice and snow competitions, understanding ice and snow sports, convenience of ice and snow venues, expensive consumption of ice and snow sports and whether 300 million people have heard of participating in ice and snow on the basis of model 1 to explain the influencing factors of cultural elites participating in ice and snow sports.

**Table 4:** Logit regression model of cultural elites participating in ice and snow sports.

	**Model 1**	**Model 2**	**Model 3**	**Model 4**	**Model 5**	**Model 6**	**Model 7**
	**Whether to participate**	**Whether to participate**	**Whether to participate**	**Whether to participate**	**Whether to participate**	**Whether to participate**	**Whether to participate**
Gender(Female = 0)	−0.141 (0.294)	−0.092 (0.303)	−0.154 (0.296)	−0.138 (0.314)	−0.16 (0.296)	−0.422 (0.326)	−0.414 (0.355)
Income	0.22 (0.161)	0.222 (0.171)	0.235 (0.162)	0.168 (0.172)	0.211 (0.161)	0.235 (0.169)	0.192 (0.18)
I like ice and snow games		0.722^***^ (0.203)					0.552^**^ (0.228)
Learn about ice and snow sports			0.226 (0.167)				0.093 (0.204)
Site convenience				0.674^***^ (0.146)			0.465^***^ (0.163)
Consumption perception					0.132 (0.207)		0.13 (0.245)
300 million people participated ice and snow						1.73^***^ (0.34)	1.65^***^ (0.363)
_cons	−0.217 (0.371)	−2.047^***^ (0.651)	−0.7 (0.517)	−0.995^**^ (0.429)	−0.353 (0.429)	−1.27^***^ (0.455)	−3.464^***^ (0.888)
Observations	215	215	215	215	215	215	215
Pseudo R2	0.007	0.054	0.013	0.091	0.008	0.106	0.187

*Standard errors are in parentheses*.

*** *p < 0.01,*

** *p < 0.05,*

* *p < 0.1*.

The estimated results of the regression equation in Table 4 show that:

1. Gender, income, and the understanding of ice and snow sports had no significant impact on the participation of cultural elites in ice and snow sports.
2. In terms of cultural factors, the number of cultural elites who like watching ice and snow competitions will increase by 1.97 times (e0.722 = 1.97) compared to those who do not like watching ice and snow competitions.
3. In terms of venue convenience, the convenience of ice and snow venues (pavilions) near the residence will increase the participation probability of cultural elite groups by 1.75 times (e0.674 = 1.75).
4. In terms of consumption perception, whether consumption is expensive has a positive correlation with participation in ice and snow, but there is no significant difference.
5. In terms of policy response, those who have heard of “300 million people participating in ice and snow sports” are 4.72 times more likely to participate in ice and snow sports than those who have not heard of it (e1.73 = 4.72).

### More or Less: An Oligit Regression Model of the Annual Number of Cultural Elites Participating in Ice and Snow Sports

The second dependent variable of this study is the continuous variable of the annual number of cultural elites participating in ice and snow sports. Therefore, ordered linear regression (ologit) is used to analyze the impact of cultural elites participating in ice and snow sports. Table 5 presents the estimation results of six sequential linear regression models on the number of ice and snow sports participating: model 1 is the basic model, and five variables are added to models 2 to 6 for model estimation, as shown in Table 5.

**Table 5:** Ologit regression model of the annual number of cultural elites participating in ice and snow sports.

	**Model 1**	**Model 2**	**Model 3**	**Model 4**	**Model 5**	**Model 6**
	**Number of participation**	**Number of participation**	**Number of participation**	**Number of participation**	**Number of participation**	**Number of participation**
Gender	0.965^**^ (0.386)	1.126^***^ (0.409)	1.43^***^ (0.425)	0.882^**^ (0.386)	0.952^**^ (0.386)	1.513^***^ (0.451)
Income	0.345 (0.214)	0.42^*^ (0.239)	0.444^**^ (0.215)	0.264 (0.221)	0.359^*^ (0.213)	0.407^*^ (0.242)
I like ice and snow games		1.364^***^ (0.288)				1.344^***^ (0.32)
Learn about ice and snow sports		−0.152 (0.237)				−0.147 (0.25)
Site convenience			0.891^***^ (0.175)			0.718^***^ (0.194)
Consumption perception				0.623^**^ (0.269)		0.839^***^ (0.294)
300 million people participated ice and snow					0.442 (0.491)	0.041 (0.581)
Observations	113	113	113	113	113	113
Pseudo R2	0.03	0.112	0.124	0.048	0.033	0.2

*Standard errors are in parentheses*.

*** *p < 0.01,*

** *p < 0.05,*

* *p < 0.1*.

The model estimation results in Table 5 show that:

1. The gender difference was significant, and the number of men participating in ice and snow sports was more significant than that of women. In model 6, with the strongest explanatory power, the possibility of increasing the number of men participating in ice and snow sports is 4.1 times (e1.51 = 4.1); even in model 4, with the lowest probability, it is 2.4 times (e0.882 = 2.4).
2. Four of the six models showed that there was a positive relationship between high income and the number of participants (Mode 2, Mode 3, Mode 5, Mode 6).
3. Interest and watching ice and snow competitions had a significant positive correlation with the number of participants participating in ice and snow sports (Mode 2, Mode 6).
4. There was no significant relationship between understanding that 300 million people have participated in ice and snow sports and the number of participants (Mode 5, Mode 6).
5. Venue convenience and consumer price had an impact on the number of participants (Mode 4, Mode 6).

## Discussion

In this study, it was seen that the interest in watching ice and snow sports, the convenience of the venue, and hearing that 300 million people are participating in ice and snow sports have a stable impact on the participation of cultural elites in ice and snow sports. This reveals that the popularization of Chinese, venues and government calls has played a positive role in promoting the development of ice and snow sports. The number of cultural elites who like watching ice and snow competitions have increased, which shows that the dissemination of ice and snow events attracts people to participate in ice and snow sports. Holding high-level ice and snow events will help guide people's initiative and enthusiasm to participate in ice and snow sports, which is also a common problem for sports events to promote sports participation (3). In this study indicated that ice and snow venues, like other sports venues, are highly related to the participation of ice and snow sports. Zhang et al. found that the improvement of snow sports facilities and venues plays a significant role in improving the attention of ice and snow sports networks (4). Also, Cheng and Xing proposed strengthening the supply of public ice and snow fitness venues to promote the upgrading of public ice and snow fitness (5). About “300 million people participating in ice and snow sports”, this study indicated that cultural elites respond very positively to the call of the party and the state. The national ice and snow sports development strategy and relevant incentive policies have played a great role in promoting Chinese society's participation in ice and snow sports.

In current study it was seen that gender had no significant impact on whether cultural elites participate in ice and snow sports. Wang et al. has been shown that pleasure and increasing ice and snow experience are the key points for female consumers to show off their ice and snow experience by ice and snow sports tourism life (6). At the same time, there was no significant difference in income, indicating that occasionally participating in ice and snow sports has no impact on their overall quality of life. Another result of the present study was that the understanding of ice and snow sports had no influence on participation in ice and snow sports. This may be because many people who participate in ice and snow sports at one time first choose to experience and have interest in tourism. This may be related to the fact that the group we choose is a culturally elite group, and there is little difference between groups in their understanding of ice and snow sports.

In terms of consumption perception, whether consumption is expensive had a positive correlation with participation in ice and snow, but there was no significant difference. This may be because for most one-time participants, occasional participation will not affect the decline of their quality of life. As Liu and Liu found in his research on the characteristics of sports consumption view of urban residents of different sizes, the sports consumption view mainly involves three aspects, such as “market environment”, “individual conditions” and “social reference” (7), and is not only concerned about the short-term quality of life.

Factors affecting the number of participants were also examined in this study. This study showed that the number of men participating in ice and snow sports was more significant than that of women. This indicated that gender inequality was in sustainable ice and snow sports participation. The reason for this inequality may be that the technical threshold of ice and snow sports is relatively high, there are certain risks in the process of participation, and it is related to the solidified female characteristics in China's social consciousness (8).

Income also affects the number of participation. This may be because the high consumption of ice and snow sports leads to the lack of economic ability of low-income groups to support their participation in ice and snow sports many times; alternatively, the higher the income is, the higher the cognitive participation of ice and snow sports (9).

The effect of interest on the number of participants indicated that watching ice and snow competitions had a significant positive correlation with the number of participants participating in ice and snow sports, which is not only consistent with the estimation results of the participation model but also confirms the research conclusion of Pan and Hai; that is, psychological internal drive can positively promote the behavior of sports tourism participants (10).

The current study showed that understanding ice and snow sports and policy responses cannot increase the number of cultural elites participating. This indicated that in the current Chinese society, the call of the government only plays a role in promoting participation, and economic strength and ice and snow cultural knowledge are still needed in sustainable participation.

The variable venue convenience and consumer price had a significant impact on the increase the annual number of cultural elites participating in ice and snow sports, and the degree of impact was also close. From another dimension, ice and snow infrastructure is an important factor in the development of China's ice and snow sports industry (11).

Increasing the physical activity level, as one of key component of the UN's 2030 Sustainable Development Goals, can have multiplicative health, social and economic benefits (1). However, there are fewer opportunities for low- and middle-income settings. In this study, it showed that income was positively correlated with the number of participants in ice and snow sports. So, people with middle- and lower incomes are less likely to participate in these sports. This lack of participation is not only a social inequality but also the influence of Olympic culture communication, which has not been brought into full play.

In general, this paper systematically analyses the factors of Chinese cultural elites participating in ice and snow sports. The main discus are as follows: first, Chinese cultural elites actively responded to the call of the Chinese government and significantly increased their participation in ice and snow sports after 2015; second, the participation of Chinese cultural elites in ice and snow sports is mostly one-time, which is related to the government's call, site restrictions and knowledge of ice and snow sports; third, the continuous participation of Chinese cultural elites in ice and snow sports is related to gender, income, ice and snow culture, venue convenience and consumption level; fourth, the government's call can only increase the participation rate, but the social promotion of ice and snow sports needs to be strengthened for sustainable participation. Therefore, to better realize the strategy of “exhibition in the south, expansion in the west and eastward” of ice and snow sports, we also need to strengthen the policy call, put forward targeted publicity strategies for the public, and improve the possibility and sustainability of the public's participation in ice and snow; cultivate ice and snow culture and build ice and snow culture according to local conditions; stimulate the ice and snow market, provide ice and snow venues and formulate sports consumption compensation mechanisms according to the consumption characteristics of ice and snow sports.

There were some deficiencies and limitations in this study. First, the survey sample of this study is targeted at the cultural elite group, which leads to group bias in the results. Therefore, in the future, we will consider adding category attributes to conduct personnel category analysis to more accurately analyze the consumption of ice and snow sports. Second, this study mainly uses the basic variables commonly used in sociological research. In the follow-up research, variables can be further added, and the promotion and participation factors and obstacles can be introduced into the regression model to explore the promotion and hindrance mechanism.

## Conclusion

Ice and snow sports are usually expensive. According to the results, low- and middle-income people are less likely to participate in these activities because of their income. Therefore, this policy can increase inequalities.

## Data Availability Statement

The raw data supporting the conclusions of this article will be made available by the authors, without undue reservation.

## Author Contributions

PL drafting the work and revising it critically for important intellectual content. WL contributed to the initial drafting of the manuscript. Both authors contributed to the article and approved the submitted version.

## Funding

This work was supported by the National Social Science Foundation of China (Grant No. 19BTY041).

## Conflict of Interest

The authors declare that the research was conducted in the absence of any commercial or financial relationships that could be construed as a potential conflict of interest.

## Publisher's Note

All claims expressed in this article are solely those of the authors and do not necessarily represent those of their affiliated organizations, or those of the publisher, the editors and the reviewers. Any product that may be evaluated in this article, or claim that may be made by its manufacturer, is not guaranteed or endorsed by the publisher.

## References

[1] World Health Organization. Global Action Plan on Physical Activity 2018–2030: More Active People for a Healthier World: at-a-glance. World Health Organization (2018). Available online at: https://apps.who.int/iris/handle/10665/272721

[2] Bao D ShuMing L. Analysis on the sustainable development path of skiing industry in Heilongjiang Province under the background of Beijing Winter Olympic Games. Front Archit Res. (2020) 2. 10.25236/FAR.2020.020210

[3] Hao S. Study on the Enlightenment of the Successful Experience of European and American Ice and Snow Sports Powers to the Development of Chinese Mass Ice and Snow Sports. Hangzhou: Hangzhou Normal University (2020).

[4] Zhang H Lingshan S Li L. Research on China's ice and snow sports network attention from the perspective of big data. Sports Sci. (2019) 40:7.

[5] Cheng W Xing L. Research on supply side governance path of demand-oriented Chinese mass ice and snow fitness. Sports Sci. (2016) 9. 10.16469/j.css.201604002

[6] Wang H Ruilin Z Ling L Wenjing Z. Study on the influencing factors of women's participation in ice and snow sports tourism. J Teach Phys Educ. (2019) 42:8.

[7] Liu W Liu X. Sociological analysis of Chinese sports viewers with differences in social capital. Complexity. (2021) 2021:8001567. 10.1155/2021/8001567

[8] Shuwang L Jiangtao M Jinglu L Ran X. Study on Influencing Factors of residents' participation in ice and snow sports in Beijing. J Capital Institute Phys Educ. (2018) 30:7.

[9] Han Q. An Empirical Study on the relationship between social stratification and physical exercise – a regression model verification based on some cities. J Guangzhou Institute Phys Educ. (2015) 35:4–9. 10.3969/j.issn.1007-323X.2015.01.002

[10] Pan J Hai L. Motivation, emotion and willingness of adventure sports tourism from the perspective of risk edge. J Shanghai Institute Phys Educ. (2020) 6:9. 10.16099/j.sus.2020.09.004

[11] Guan C. Realization of the goal of “300 million people participating in ice and snow sports” under the background of 2022. Beijing Winter Olympic Games. Ice Snow Sports. (2017) 39:4.

